# SPC24 is critical for anaplastic thyroid cancer progression

**DOI:** 10.18632/oncotarget.15670

**Published:** 2017-02-24

**Authors:** Huabin Yin, Tong Meng, Lei Zhou, Haiyan Chen, Dianwen Song

**Affiliations:** ^1^ Department of Orthopedics, Shanghai General Hospital, School of Medicine, Shanghai Jiaotong University, Shanghai, 200080, China; ^2^ Department of Bone Tumor Surgery, Changzheng Hospital, The Second Military Medical University, Shanghai, 200003, China; ^3^ Department of Rheumatology, Shanghai Guanghua Hospital of Integrated Traditional and Western Medicine, Shanghai, 200052, China; ^*^ These authors have contributed equally to this work

**Keywords:** SPC24, metastasis, thyroid cancer

## Abstract

In the past 2 decades, the incidence of thyroid cancer has been rapidly increasing worldwide. Anaplastic thyroid cancer (ATC) is the most lethal of all thyroid cancers and one of the most aggressive human carcinomas. SPC24 is an important component of the mitotic checkpoint machinery in the tumorigenesis and high levels of SPC24 have been found in colorectal and hepatocellular carcinomas, but its role in anaplastic thyroid cancer is still unclear. Our results showed that SPC24 was high expressed in human thyroid cancer samples. In addition, knockingdown endogenous SPC24 could repress cell growth, inhibit cell invasive ability and promote apoptosis in different ATC cells. Next, *in vivo* xenograft studies indicated that the SPC24 knockdown cells has decreased tumor size compared to the controls. This conclusion is also endorsed by our studies using human thyroid cancer samples. Taken together, our data demonstrates that SPC24 can serve as a promising prognostic biomarker of ATC cells and it is a novel strategy which could be developed by targeting SPC24 in future.

## INTRODUCTION

In the past 2 decades, the incidence of thyroid cancer has been rapidly increasing worldwide [1, 2].

Anaplastic thyroid carcinoma (ATC) accounts for 1–2% of all thyroid malignancies. Although ATC is rare, it is one of the most aggressive human cancers and the average survival time of ATC is only 6 to 8 months and the 5-year survival rate of ATC is only 0 ∼ 10% [3, 4]. Most patients die due to tumor invasion or distant metastases. Therefore, the identification of novel therapeutic molecules for ATC may help understanding the pathogenesis of the disease and improving the outcome of the patients.

The four-protein Ndc80 complex comprised of Ndc80 (also called Hec1 or KNTC2), Nuf2, SPC24 and SPC25, which together form a dumbbell-like heterotramer [5]. Nuclear division cycle 80 (Ndc80) is essential for the stable formation of kinetochore-microtubule anchoring and correct chromosome segregation during mitosis [6]. The Ndc80 complex directly mediates microtubule binding by the Ndc80/Nuf2 heterodimer [6–8], and the SPC24 and SPC25 heterodimer anchor the Ndc80 complex to the inner kinetochore [6, 9]. It was reported that simultaneous disruption of both SPC24 and SPC25 genes rendered the cell to be spindle checkpoint defective, which allowed the cell to bypass mitosis [10]. Furthermore, high levels of SPC24, CDCA1 and SPC25 were correlated with colorectal and hepatocellular carcinoma tumors [6, 11], suggesting the potential roles of this protein in cancer development. However, the function of SPC24 in ATC remains unclear.

Metastasis is the spread of tumor cells to tissues and organs other than where it is originated and the formation of new tumors [12]. E-cadherin is a cell adhe-sion transmembrane molecule which is a member of a family of functionally related transmembrane glycopro-teins that mediates Ca2+-dependent intercellular cellular adhesion [13]. The treat-ment options for ATC include surgery, chemotherapy and radiotherapy. Effective strategies for the prevention of ATC metastasis are still lack because the rare incidence of ATC and its aggressive nature [14, 15]. The development of effective curative treatments for patients with ATC has been hampered by its relative rarity [16]. Another major hurdle in this endeavor has been the lack of an appropriate animal model in which potential therapeutic strategies against ATC may be evaluated [16].

In the present study, we have attempted to investigate the effects of SPC24 deficiency on ATC cell proliferation and survival *in vitro* and *in vivo*. We indicated that knockdown of SPC24 inhibited the proliferation, and promoted cell apoptosis in ATC cells. Moreover, knocking down SPC24 increased the expression of E-cadherin and inhibited cell migration and invasion of anaplastic thyroid cancer cells. In order to better understand its mechanisms of tumor progression, we have developed an *in vivo* xenograft model of ATC in nude mice and the results showed that SPC24 promoted tumour development. This conclusion was also endorsed by our studies using human thyroid cancer samples. Taken together, these findings indicated that SPC24 served as a prognostic cancer biomarker of ATC by downregulating E-cadherin. Thus, SPC24 may serve as a novel therapeutic target for ATC treatment.

## RESULTS

### SPC24 was high expressed in human thyroid cancer samples

**Figure 1:**
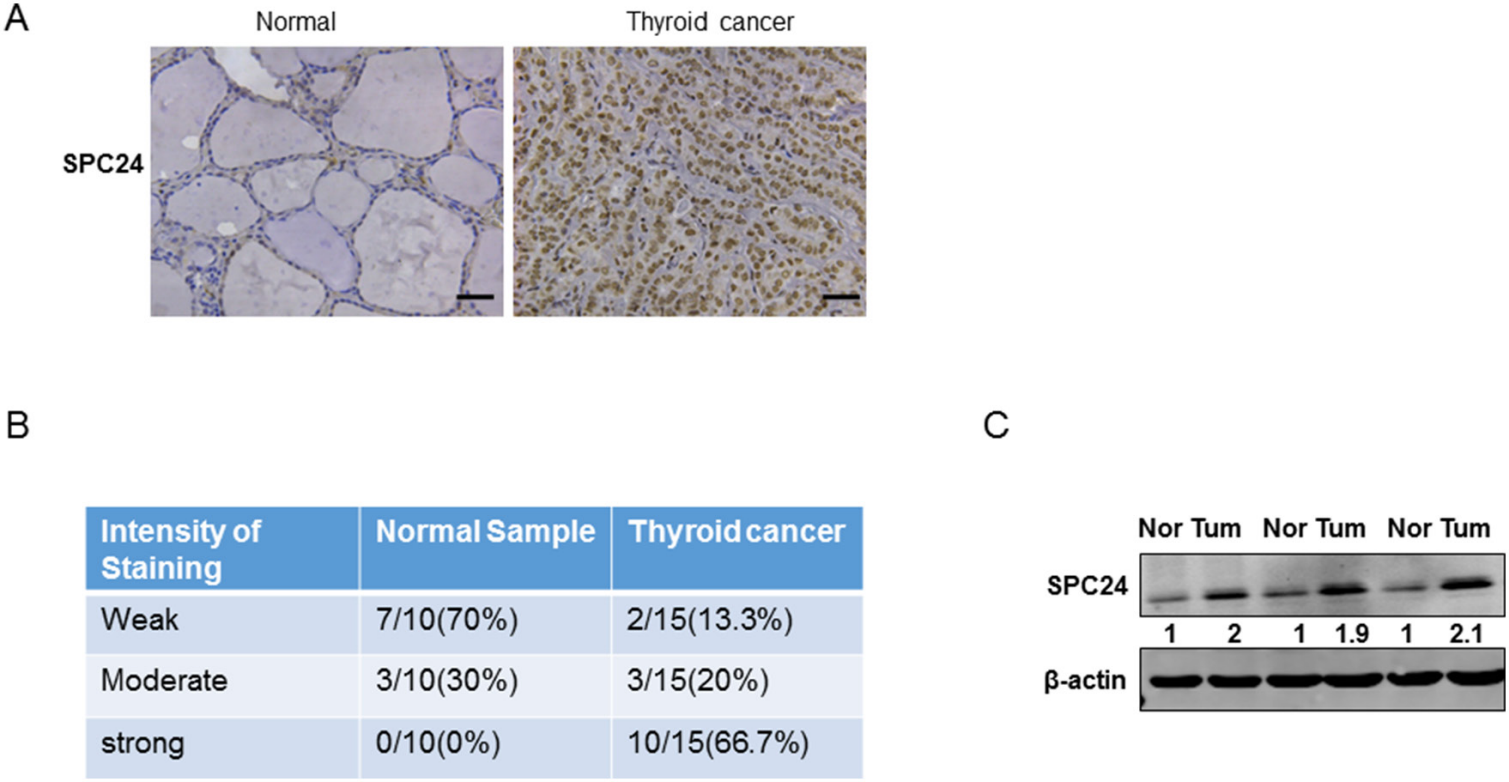
SPC24 was high expressed in human thyroid cancer samples **(A)** IHC analysis of SPC24′s expression in the human thyroid cancer samples (Scale bar, 50μm; original magnification, ×20). **(B)** The semiquantitative analysis of SPC24 immunohisochemical staining in human normal and thyroid cancer samples. **(C)** Immunoblot analysis of SPC24 expression in different normal and human ATC samples.

To evaluatehe role of SPC24 in ATC progression, the expression of SPC24 was first tested in human thyroid cancer samples. As shown in Figure 1A, the immunohistochemical analysis of human thyroid cancer samples indicated that the expression of SPC24 was high expressed compared to the normal samples. To further confirm these findings, we used Western Blotting assay to define the same phenomenon as IHC results (Figure 1B). Statistical analysis endorsed that high levels of SPC24 was detected in human thyroid cancer samples (Figure 1C).

### Silence of endogenous SPC24 by siRNA suppressed the proliferation of ATC

**Figure 2:**
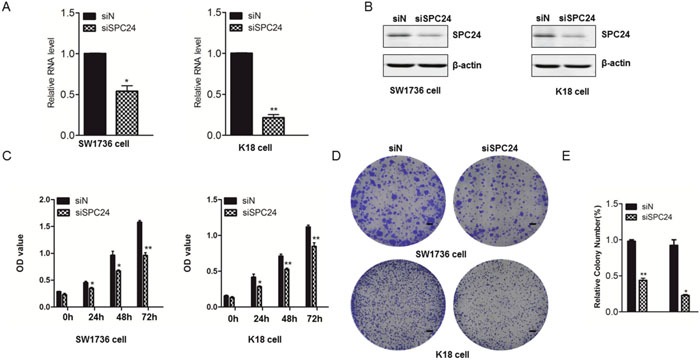
Silence of endogenous SPC24 by siRNA suppressed the proliferation of ATC **(A)** and **(B)** SW1736 and K18 cells were transfected with siRNA against SPC24 or the negative control (non-targeting) siN for 72 hours. Real time PCR **(A)** or western blot analysis **(B)** was done in SW1736 and K18 cells. Actin serves the loading control. **(C)** After 72 hours post tansfection with siRNA against SPC24 or the negative control (non-targeting) siN, SW1736 and K18 cell viability was monitored by MTT assay at the indicated times. **(D, E)** Soft-agar colony formation for SW1736 and K18 cells transfected with scrambled siRNA (negative control, siN) and SPC24-siRNA, respectively. (Scale bar, 100μm; original magnification,×10). *p < 0.05, **p < 0.01.

The role of SPC24 during ATC progression was detected in ATC cells. To investigate the effect of SPC24 on cell growth, we slienced SPC24 in SW1736 and K18 cells using siRNA. The results were demonstrated by semi-quantitative RT–PCR (Figure 2A) and Western Blotting (Figure 2B) in ATC cells. Next, MTT assay showed that the cell growth of SW1736 and K18 was inhibited after the knockdown of SPC24, suggesting that SPC24 could significantly promote cell growth. (Figure 2C). To further define the prognostic role of SPC24 on ATC cell growth, we did crystal violet staining assay and the results suggested that SPC24 knockdown negatively regulated cell proliferation in ATC cells (Figure 2D, 2E)

### Downregulation of SPC24 induced the cell cycle arrest and promoted cell apoptosis

**Figure 3:**
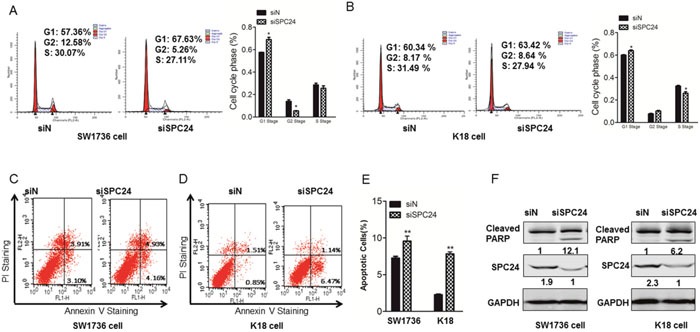
Downregulation of SPC24 induced the cell cycle arrest and promoted cell apoptosis **(A)** and **(B)** Cell cycle analyses were performed in SW1736 cells **(A)** and K18 **(B)** cells transfected with scrambled siRNA (negative control) and SPC24-siRNA by flow cytometry. **(C)** and **(D)** SW1736 and K18 cells were collected and analysed for annexin-V and PI labelling. The percentage of cells that were labelled with annexin-V (primary apoptosis) and with annexin-V plus PI (secondary apoptosis) was measured. The fraction of the total percentages that showed only annexin-V labeling and that showed annexin-V plus PI labelling are shown. **(E)** Quantify analysis of the apoptotic cells was shown in SW1736 and K18 cells transfected with siRNA and SPC24-siRNA. **(F)** Cells were treated with scrambled siRNA (control) or SPC24 siRNA for 72 hrs and then collected for Western Blot analysis. Cells were lysed and analysed by immunoblotting with the antibodies against PARP, and SPC24. *p < 0.05, **p < 0.01.

SPC24 is an important component of the mitotic checkpoint machinery in the tumorigenesis. Therefore, we analyze the cell cycle of ATC. Flow cytometry analysis showed that the percentages of cells in G1-phase were obviously increased in SPC24 knockdown cells (Figure 3A, 3B). Moreover, analysis of apoptotic cells by flow cytometry revealed that knocking down SPC24 led to a significant increase of the percentage of annexin V-positive fractions in SW1736 and K18 cells (Figure 3C, 3D). The quantify analysis showed the same trend as flow cytometry analysis (Figure 3E). We also detected PARP in ATC cells because cleaved PARP is one of the characteristic symbols of cell apoptotic process. As expected, the cleaved PARP was markedly increased after SPC24 silencing compared with their control groups (Figure 3F). These results collectively suggested that SPC24 played its role in oncogenesis.

### SPC24 increased ATC cell invasive ability and mesenchymal attributes

**Figure 4:**
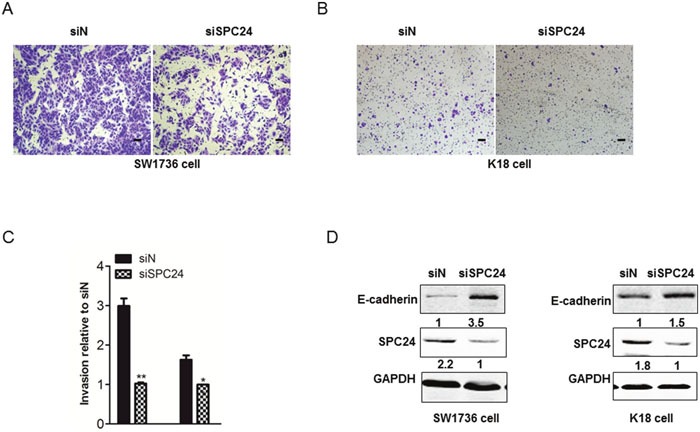
SPC24 increased ATC cell invasive ability and mesenchymal attributes **(A)** and **(B)** SW1736 and K18 cells were transduced with SPC24 siRNA or scrambled siRNA and 72h post-infection a transwell invasion assay was performed. The cells were allowed to migrate for 24h at 37°C. Images were captured by a digital camera and magnified by ×250. (Scale bar, 100μm; original magnification, ×10). **(C)** Quantitative assay were performed in SW1736 and K18 cells transduced with SPC24 siRNA or scrambled siRNA. **(D)** Cells were treated with SPC24 siRNA for 72 hrs and then collected for Western Blot analysis. Cells were lysed and analysed by immunoblotting with the antibodies against E-cadherin, and SPC24. *p < 0.05, **p < 0.01.

EMT is the key step in cancer metastasis and the decreased expression of E-cadherin is the inducer of EMT, we further study whether SPC24 is involved in regulating cell migration. The results showed that the invasion ability markedly decreased in SW1736 and K18 cells after silencing SPC24 (Figure 4A/4B). Quantified analysis for cell invation showed the same phenomenon in SW1736 and K18 cells (Figure 4C). In addition, we examined the expression of E-cadherin in ATC cells. Indeed, the western blot experiments showed that the expression of E-cadherin was dramatically increased in SW1736 and K18 cells transfected with SPC24 siRNA compared with that in control siRNA-treated cells (Figure 4D). These results indicated that SPC24 increased ATC cell invasive ability by downregulating E-cadherin.

### SPC24 promoted tumor initiation in xenograft mouse model and in human samples

**Figure 5:**
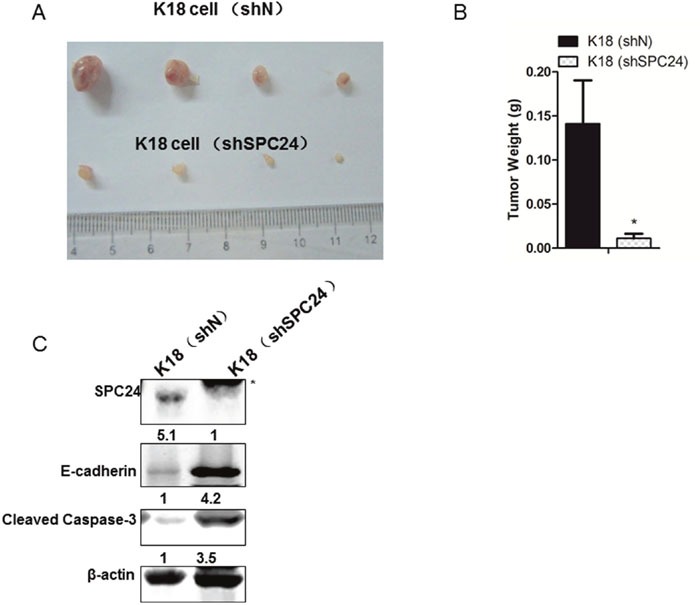
SPC24 promoted tumor initiation in xenograft mouse model and in human samples **(A)** Effects of SPC24 downregulation on growth of K18 subcutaneous xenografts *in vivo*. Nude mice were injected with 2×10^6^ cells and tumors were removed at three weeks post injection; each experimental group contained 4 tumors. **(B)** The weight of tumors in shSPC24 cells was markedly reduced in comparison with that in shN cells. **(C)** IHC analysis of SPC24 and E-cadherin expression in the mice tumor tissues. Asterisk (*) refers to non-specific bands. *p < 0.05.

**Figure 6:**
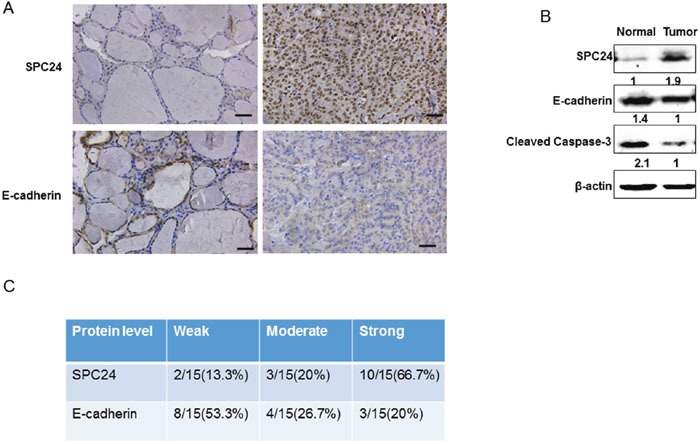
SPC24 was high expressed and E-cadherin was downregulated in human thyroid cancer samples **(A)** IHC analysis of SPC24 and E-cadherin expression in the human thyroid cancer samples (Scale bar, 50μm; original magnification, ×20). **(B)** Immunoblot analysis of SPC24, Cleaved Caspase-3 and E-cadherin expression in normal and human ATC samples. An anti-β-actin immunoblot is shown as the loading controls. **(C)** The semiquantitative analysis of SPC24 and E-cadherin immunohisochemical staining in human thyroid cancer samples.

To analyze the effects of SPC24 knockdown on ATC cell growth *in vivo*, we subcutaneously injected 2×10^6^ K18-shN and shSPC24 cells in two flanks of BALB/C nude mice. As shown in Figure 5A/5B, the volume and the weight of tumors in shSPC24 cells was markedly reduced in comparison with that in shN cells, suggesting that SPC24 functions as an tumor inducer in K18 cells. Furthermore, we also detected the expression of E-cadherin and the results indicated that the level of E-cadherin increased in SPC24 knockdown tumors. Caspase3 activity which was another characteristic symbol of cell apoptotic process increased in SPC24 knockdown tumors indicating SPC24 functions as an oncogene (Figure 5C). This conclusion was also endorsed using human thyroid cancer samples by IHC staining and Western Blotting assay (Figure 6A/6B). Quantified analysis for IHC of human thyroid cancer samples showed SPC24 was negatively associated with E-cadherin (Figure 6C).

## DISCUSSION

Anaplastic thyroid carcinoma (ATC) is a rare thyroid malig-nancy, accounting for less than 2% of all cases of all thyroid cancers and it is often lethal. Since anaplastic thyroid cancer (ATC) doesn't respond to radioiodine, radiotherapy or chemotherapy, and new therapeutic approaches are needed. The high mortality of thyroid cancer is largely attributed to early metastasis and most patients die due to the invasion or distant metastases. Therefore, the identification of novel therapeutic molecules for ATC may help understanding the pathogenesis of ATC.

The changes in chromosomal copy number (aneuploidy) and chromosome mis-segregation have been recognized as one of the most noticeable traits of cancers, which contribute directly to the development and metastasis of malignant tumor [6, 17, 18]. As a core component of the Ndc80 kinetochore complex, SPC24 is essential for chromosomal directional movement to the spindle poles in anaphase [19]. This is the first study demonstrating that SPC24 is involved in the regulation of E-cadherin expression in anaplastic thyroid carcinoma. In studying the role of SPC24 in anaplastic thyroid cancers, we demonstrated that silence of endogenous SPC24 by siRNA suppressed the proliferation of ATC. In addition, we found that downregulation of SPC24 induced the cell cycle arrest and promoted cell apoptosis.

The previous studies demonstrated SPC24 might be closely associated with the progression, invasion and metastasis of HCC [6]. And we all know that EMT is the key step in cancer metastasis [20]. Therefore, we showed that SPC24 increased ATC cell invasive ability by downregulating E-cadherin. Our *in vivo* studies revealed SPC24 promoted tumor initiation and this was the direct proof for the function of SPC24 in anaplastic thyroid cancer progression. Furthermore, the clinicopathologic/prognostic significance of SPC24 was also detected in clinical samples. We demonstrated that the protein level of SPC24 combined with decreased E-cadherin was significantly increased in human ATC samples. In our study, we provide the first evidence that SPC24 functions as a tumor initiator for ATC prgression. However, additional studies are necessary to elucidate the cross-talk between SPC24 and the EMT signalling pathway during anaplastic thyroid carcinogenesis in larger patient samples or the transgenic mice.

In summary, this study provides a mechanistic understanding of SPC24 action on ATC cells. Our *in vitro* and *in vivo* studies are the first to demonstrate that inhibition of SPC24 significantly attenuates the survival and proliferation of ATC cells, substantiating that SPC24 functions as an oncoprotein to activate EMT pathway via downregulating E-cadherin. All these results suggest that silencing SPC24 play a protective role in anaplastic thyroid cancer and can be targeted for the development of novel diagnostic and therapeutic strategies.

## MATERIALS AND METHODS

### Cell culture

SW1736 and K18 cells were purchased from ATCC. The stable cell lines were generated by integration of retroviral shRNA vectors specific for SPC24 or a control gene from OriGene (Rockville, MD).

### siRNA transfection

The transfection reagent TransExcellent-siRNA was purchased from Cenji Biotech. (Shanghai, China) 2 ug of total RNA was transcribed into cDNA with M-MLV reverse transcriptase (Invitrogen) following the manufacturer's instruction. The cDNA was amplified by PCR using specific primer pairs for SPC24. The siRNA against SPC24 shown as follows was described previously [19]. The siRNA sequences are 5′-GAGCCUUCUCAAUGCGAAGTT-3′ which was described previously [6].

### MTT assay

Cells were seeded in 96-well microplates and incubated for 24 h. The cells were then treated control or SPC24 siRNA for 72 hr. MTT [100 μl (5 g/l)] was added to the cells which were then cultivated for another 4 hr. The absorbance was measured at 570 nm by an ELISA reader.

### Transwell assay

Cell invasion was measured by using the Matrigel-coated transwell culture chambers as described previously [21]. SW1736 and K18 cells were prepared in serum-free medium and were transfected with control or SPC24 siRNAs and plated in the upper chamber of transwell chambers. The lower chamber was filled with 10% FBS-containing medium. The invasive cells penetrated through the Matrigel in the lower chamber were fixed and photographed using a light microscope for quantification.

### Xenograft animal model

The female BALB/c nude mice at the age of 5 weeks were anesthetized and the K18 stable cell lines (shN/shSPC24) were implanted into the dorsal flanking sites of nude mice at 2×10^6^ cells in 100 μl per spot. Three weeks after injection, mice bearing tumors were killed for the assessment of tumor size and immunohistological examination.

### Immunohistochemistry (IHC)

Tissues were prepared from paraffin-embedded blocks. Sections were incubated with antibodies indicated in this study for overnight in a humidified container at 4°C. Next, the slides were incubated with the secondary antibody conjugated with horseradish peroxidase was performed for 1 hr at room temperature. Sections were finally stained with 3, 3-diaminobenzidine tetrahydrochloride (DAB) and counterstained with hematoxylin.

### Statistical analysis

Data are presented as mean ± s.e.m. of three independent experiments. Significance of means between two groups is determined by student's t-test or two way ANOVA. A P-value of < 0.05 was considered significantly different.
